# ONDL: An optimized Neutrosophic Deep Learning model for classifying waste for sustainability

**DOI:** 10.1371/journal.pone.0313327

**Published:** 2024-11-08

**Authors:** Nour Eldeen Mahmoud Khalifa, Mohamed Hamed N. Taha, Heba M. Khalil, Mazhar Hussain Malik

**Affiliations:** 1 Information Technology Department, Faculty of Computers and Artificial Intelligence, Cairo University, Giza, Egypt; 2 Computer Science Department, Faculty of Computers & Artificial Intelligence, Benha University, Benha, Egypt; 3 School of Computing and Creative Technologies College of Arts, Technology and Environment (CATE), University of the West of England Frenchay Campus, Bristol, United Kingdom; National Institute of Technology Rourkela, INDIA

## Abstract

Sustainability has become a key factor on our planet. If this concept is applied correctly, our planet will be greener and more eco-friendly. Nowadays, waste classification and management practices have become more evident than ever. It plays a crucial role in the sustainability ecosystem. Computer algorithms and deep learning can help in this sustainability challenge. In this paper, An Optimized Neutrosophic Deep Learning (ONDL) model was proposed to classify waste objects. Two datasets were tested in this research {Dataset for Waste Management 1 (DSWM1), and Dataset for Waste Management 2 (DSWM2)}. DSWM1 consists of two classes (Organic or Recycled) objects. The DSWM2 consists of three classes (Organic, Recycled, or Non-Recyclable) objects. Both datasets exist publicly on the internet. The ONDL model architecture is constructed based on Alexnet as a Deep Transfer Learning (DTL) model and the conversion of images to True (T) neutrosophic domain and Grey Wolf Optimization (GWO) for the image features selection. The selection process of the building components of the ONDL model is comprehensive as different DTL models (Alexnet, Googlenet, and Resnet18) are tested, and three neutrosophic domains (T, I, and F) domain are included. The ONDL model proved its efficiency against all the tested models, moreover, it achieves competitive results with related works in terms of testing accuracy and performance metrics. In DSWM1, the ONDL model achieved 0.9189, 0.9177, 0.9176, and 0.9177 in Testing Accuracy (TA), Precision (P), Recall (R), and F1 score. In DSWM2, it achieved 0.8532, 0.7728, 0.7944, and 0.7835 in TA, P, R, and F1 Score consequently.

## 1 Introduction

Sustainability refers to the practice of fulfilling the demands of the current generation while ensuring that the capacity of future generations to fulfill their own needs remains intact. Achieving a balanced equilibrium between economic expansion, ecological conservation, and societal welfare is integral to this endeavor [1].

The global production and accumulation of waste have reached billions of metric tons every year [2]. This observed phenomenon can be recognized by the growing prevalence of urbanization, whereby a larger population resides in major metropolitan centers, resulting in a proportional rise in waste generation stemming from home, commercial, and industrial endeavors. The issue of waste management presents a global challenge, encompassing a multitude of environmental considerations [3].

A portion of these waste materials are deposited in landfills, where they are interred to facilitate gradual decomposition. Additionally, a portion of these waste materials is subjected to incineration as a result of the mounting pressure exerted by the substantial volume of waste materials on the finite capacity of landfills [4]. Certain types of waste are commonly disposed of in soil, oceans, and rivers, particularly in less developed nations where waste management systems are less advanced. The accumulation of solid waste in metropolitan areas is a major worry that, if not effectively handled, could lead to environmental pollution and be dangerous to human health. To manage a range of waste products, it’s crucial to have an advanced/intelligent waste management system. The process of separating waste into its various components is one of the most crucial parts of waste management, and it is typically carried out manually by hand-picking [5].

The primary objective of sustainable waste management is to optimize resource utilization through the prolonged retention of materials in active circulation. Furthermore, its primary objective is to prioritize the reduction of solid waste that is deposited in landfills or incinerated. There is a pressing need for the implementation of novel waste management strategies to effectively address current waste streams and concurrently reduce overall waste generation [6].

The field of sustainability research with Artificial Intelligence (AI) places significant emphasis on the utilization of machine learning (ML) models and algorithms to demonstrate the capacity of machines to analyze and acquire knowledge from data. Machine learning (ML) encompasses a range of approaches, such as reinforcement learning, transduction, and multitasking, as well as supervised, unsupervised, and semi-supervised learning [7]. Deep Learning is widely recognized as a subfield of machine learning that relies on algorithms to facilitate data processing and simulate cognitive processes, as well as to construct abstractions. Deep Learning employs a hierarchical approach to transform input data into output predictions, leveraging multiple layers of computational approaches to effectively analyze and discern latent patterns within the data, particularly in the context of visual object detection. In a deep network, information is propagated via successive layers, where the output of each layer serves as the input for the subsequent layer. The initial layer in a deep neural network is referred to as the input layer, whilst the last layer in the deep network is known as the output layer. The hidden layers are located intermediate to the input layers and output layers [8]. Deep learning is used in many domains such as Visual Question Answering (VQA) [9, 10], potato leaf disease classification, plant leaves disease recognition, and date classification [11–13], semantic segmentation for COVID-19 lesions [10], the detection of COVID-19 in CT medical images [14, 15], COVID-19 infections predictions models [16], and waste classifications [17–19].

Also, Neutrosophic can help in sustainability applications. Neutrosophic is a mathematical paradigm that addresses the concepts of indeterminacy, incompleteness, and inconsistency within decision-making processes. The conventional binary logic, which operates based on true or false values, is expanded by the inclusion of a third truth value known as "indeterminacy" which serves to represent information that is uncertain or unclear [20]. Neutrosophic logic demonstrates significant use in addressing intricate and unpredictable systems, rendering it usable across diverse domains, such as COVID-19 X-ray classification [20, 21], face mask classification [22], and waste management. The main contributions of this research are:

1. We propose a new model, ONDL, for waste classification based on Alexnet as a Deep Transfer Learning (DTL) model and the conversion of images to True (T) neutrosophic domain and Grey Wolf Optimization (GWO) for the image features selection.
2. A new features selection layer was added to customize the architecture of the Alexnet model. This layer is based on GWO for the feature selection process. This layer enhances the model’s efficiency and performance by selecting the most relevant features.
3. Investigation of different DTL models on different Neutrosophic domains.
4. We have compared the new proposed ONDL model with the original Alexnet model and other related works, the ONDL achieved competitive results against the Alexnet model, other DTL models, and related works in terms of testing accuracy, performance metrics, number of selected features, consumed training time, and testing time.

The forthcoming sections of this paper will explore various aspects of the proposed model. Section 2 will present a comprehensive review of the relevant literature. Section 3 will provide an overview of the dataset characteristics. The process of selecting the ONDL model will be elaborated upon in Section 4. In the subsequent section, Section 5, the experimental results that have been acquired will be presented. Lastly, the research will conclude with Section 6, wherein potential opportunities for future research will also be outlined.

## 2 Related works

Previous studies on waste classification have investigated diverse methods and approaches for the efficient identification and categorization of waste objects. In [23], The authors proposed a waste classification approach that constructively utilizes deep learning. The dataset comprises various categories of waste materials, which are metals, non-recyclables, paper, unknown waste, plastics, vital waste, and glass. The researchers employed Efficientnet [24] in their study, and their model achieved a mean accuracy of 0.75 in classification for the test dataset.

The authors in [25], The researchers utilized the TrashNet dataset to train and assess different deep-learning architectures for the automated categorization of waste categories. Significantly, a comparative analysis was performed on various Convolutional Neural Network (CNN) architectures, specifically VGG, Inception, and ResNet. Among the various models considered, the combined Inception-ResNet architecture demonstrated the most positive performance, attaining a notable accuracy rate of .8806 in the task of classification.

In the fields of sustainability and waste classification, there are not enough research studies that have employed the same dataset used in our research. In [26], the authors proposed a five-layer CNN for Solid Waste Image Classification. The authors used data augmentation to avoid overfitting. The achieved accuracy was 0.8088. The authors in [17] developed a new framework for efficient future waste management. To enhance the accuracy and effectiveness of waste classification, a novel learning approach (Learning method with a deep neural network for smart systems) was proposed. This method utilizes deep neural networks to achieve superior performance in waste classification. The proposed framework surpassed the AlexNet, VGG16, and ResNet34 models by achieving an accuracy of 0.9453. The authors of [27] suggested a new method for classifying waste into recyclable, non-recyclable, and organic materials by using neural networks to identify waste objects. Training accuracy for the model was 0.8377, and testing accuracy was 0.8125. Table 1 presents a comparison of related works that use the same dataset used in our study of not.

**Table 1:** Comparison of related works that use the same dataset used in our study of not.

Ref	The same Dataset in our study	Year	Model	Accuracy	Advantages	Disadvantages
[25]	No	2019	Inception-ResNet	0.8806	Comparative analysis of various CNN architectures, achieving high accuracy.	The study is limited to the TrashNet dataset and may not generalize well to other waste classification scenarios.
[27]	Yes	2020	CNN	0.8125	Focuses on classifying waste into recyclable, non-recyclable, and organic categories.	The reported accuracy is relatively lower compared to other state-of-the-art methods.
[23]	No	2022	EfficientNet	0.7500	Constructive use of deep learning for waste classification.	The dataset may not be diverse enough for real-world applications.
[26]	Yes	2022	5-layer CNN	0.8088	Uses data augmentation to prevent overfitting.	The model architecture is relatively simple and may not capture complex features in waste images.
[17]	Yes	2023	LADS (deep neural network)	0.9453	Novel learning approach for smart systems, outperforming several established models.	The study lacks details on the specific architecture and implementation of the LADS method.

The ONDL model proposed in this paper contributes to addressing some of these disadvantages. By incorporating neutrosophic image conversion and GWO-based feature selection, the ONDL model enhances the efficiency and accuracy of waste classification. The neutrosophic conversion improves the representation of waste objects, while GWO reduces the dimensionality of the feature space, leading to a more computationally efficient and robust model. The model’s ability to achieve high accuracy on both DSWM1 and DSWM2 datasets, even with their inherent class imbalance, demonstrates its potential to address real-world waste classification scenarios.

## 3 Dataset characteristics

Two datasets are investigated in this research {Dataset for Waste Management 1 (DSWM1) [28], and Dataset for Waste Management 2 (DSWM2) [29]}. The DSWM1 is a dataset with open access. It is comprised of 25,077 images of waste objects and is divided into two categories, Organic (O) and Recyclable (R) objects. The O class contains 13,966 samples, while the R class contains 11,111. The dataset consists of cardboard, glass, metal, paper, plastic, fabric, and organic consumables. The image resolutions are not identical. The images have a JPG file extension. The images within the dataset have been gathered from the Google Images and ImageNet websites as presented in Table 2.

**Table 2:** Datasets attribute description.

Dataset Name	Number of Images	Classes	Class Distribution	Image Types
DSWM1 [28]	25,077	Organic (O), Recyclable (R)	O: 13,966	Cardboard, metal, glass, paper, plastic, fabric, organic consumables
			R: 11,111	
DSWM2 [29]	25,509	Organic (O), Recyclable (R), Non-recyclable (N)	O: 14,001	Mirrors, window glass, light bulbs (in addition to DSWM1 types)
			R: 8,264	
			N: 3,244	

**Fig 1:**
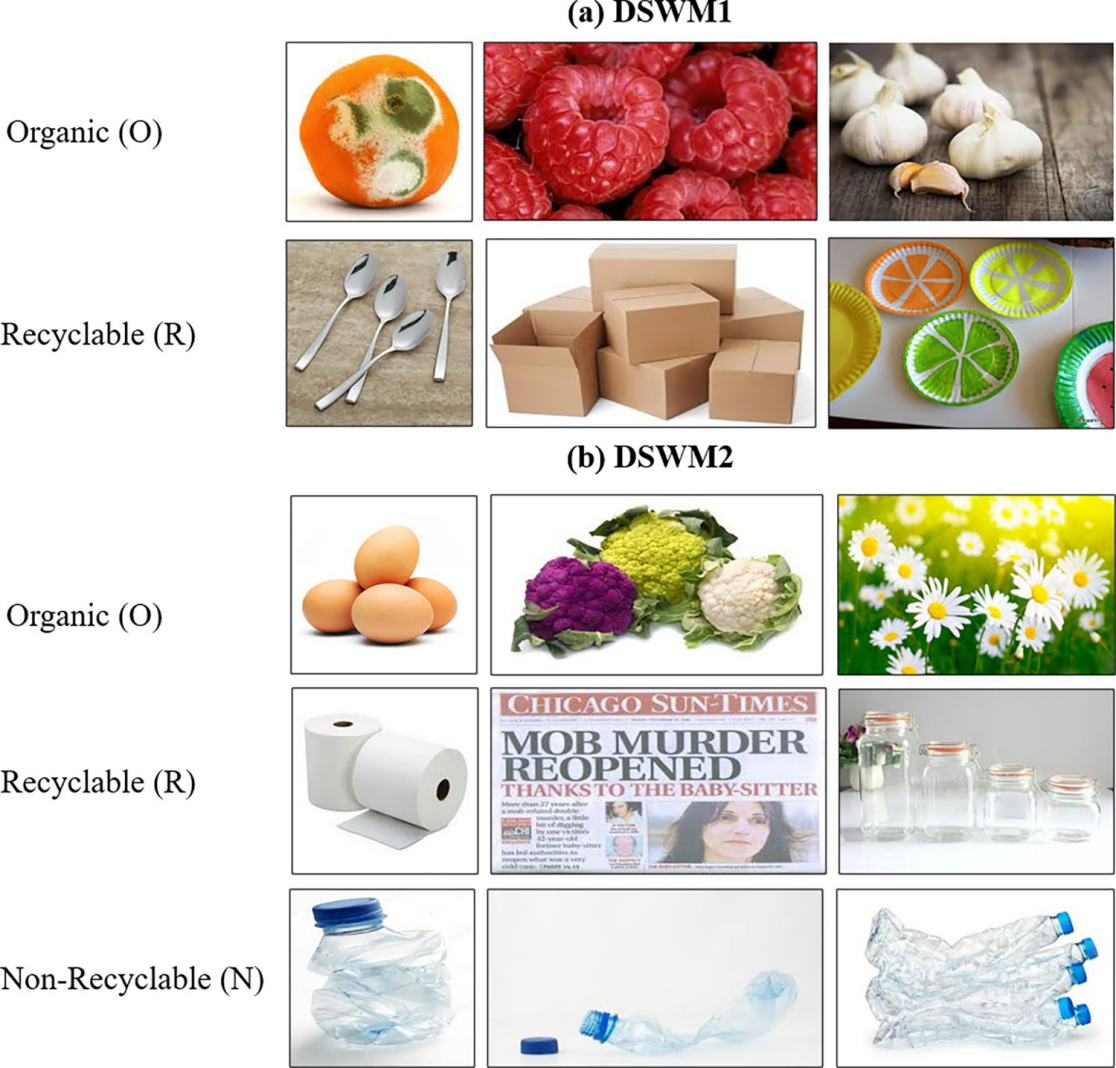
Dataset image samples for (a) DSWM1 [28], and (b) DSWM2 [29].

The DSWM2 is a dataset with open access too. It is a continuation of DSWM1. It consists of 25,509 images of waste objects categorized as Organic (O), Recyclable (R), and Non-recyclable (N) objects. The O class contains 14001 samples, while the R class has 8264 and the N class has 3244. The (N) class contains mirrors, window glass, and light bulbs. The images within the dataset have been gathered from the Google Images and ImageNet websites. Fig 1 illustrates DSWM1 and DSWM2 samples.

One of the potential biases is image resolutions in DSWM1 and DSWM2 are not identical, which could introduce variability in the quality and clarity of the images, potentially affecting the performance of the deep learning models. Additionally, both datasets exhibit class imbalance, where the ’Organic’ class has a significantly higher number of samples compared to the other classes. This imbalance can bias the models towards the majority class, leading to reduced accuracy for the minority classes. We solved those challenges by changing all images to a standard resolution of 72 pixels per inch (PPI). The severity of the class imbalance, data augmentation, and resampling were used.

## 4 Proposed model selection process

**Fig 2:**
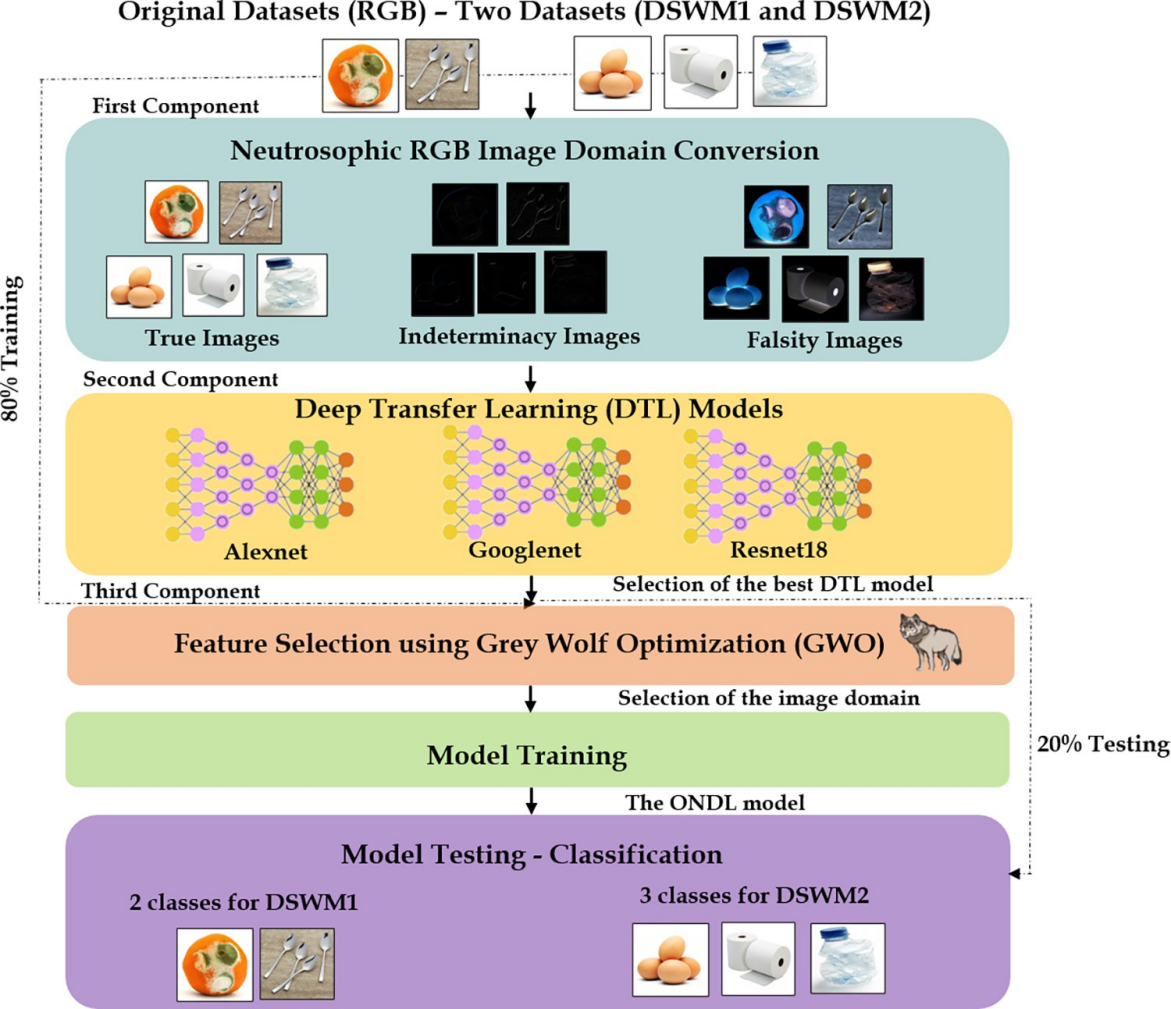
The selection process of the ONDL model among other models.

The proposed model {Optimized Neutrosophic Deep Learning Model (ONDL)} selection process includes the selection of its building components. ONDL was built based on three components. The first component is the conversion to RGB neutrosophic domain for dataset images. The second component is Deep Transfer Learning Models (DTL) to extract image features and for classification. The third component is feature selection using Grey Wolf Optimization (GWO). Fig 2 presents the selection process of the ONDL model among other models.

The selection and the nomination process of the ONDL model is presented in Algorithm 1. The ONDL model selection algorithm focuses on choosing the most appropriate deep transfer learning (DTL) model and image domain for training, using images from two datasets (DSWM1 and DSWM2). The algorithm begins by initializing several variables: the best model, the best image domain, and the highest test accuracy (TA) are set to default values. Three DTL models (AlexNet, GoogleNet, and ResNet18) and four image domains (RGB, True Neutrosophic, Indeterminacy Neutrosophic, and Falsity Neutrosophic) are used. For each combination of a model and image domain, the algorithm preprocesses the images and splits the dataset into training and testing sets. It then augments the training data and trains the model using a specific set of hyperparameters (learning rate, epochs, batch size, and early stopping). After training, the model is evaluated based on test accuracy, precision, recall, F1 score, and execution time.

The most appropriate model and image domain combination is selected based on the highest test accuracy (TA). If two combinations achieve the same accuracy, the model with the lower training and testing time is preferred. Once the best model and domain are identified, a feature selection method, Grey Wolf Optimizer (GWO), is applied to enhance the selected model’s performance. The ONDL model is then trained using the best model, the selected features, and the training data. Finally, the model is evaluated once more on the test set, with the final test accuracy, precision, recall, and F1 score recorded. This iterative process ensures the selection of an optimal model and image domain for the ONDL system.

**Algorithm 1.** The selection process algorithm of ONDL model

**Input:** Images from two datasets (DSWM1 and DSWM2

**Output:** The ONDL model

**best_model** = None

**best_domain** = None

**best_TA** = 0

**best_time** = 0

**DTL_models** = [Alexnet, Googlenet, Resnet18]

**image_domains** = [RGB, True(T) Neutrosophic, Indeterminacy(I) Neutrosophic, Falsity(F) Neutrosophic]

**foreach** ∀model ∈ DTL_models do

    **foreach** ∀domain ∈ image_domains do

        **processed_images** = preprocess_images(images, domain)

        X**_train, X_test, y_train, y_test** = train_test_split(processed_images, labels, test_size = 0.2)

        **X_train** = augment_data(X_train)

        **clf** = train_DTL_model(model, X_train, y_train, learning_rate = 0.001, epochs = 50, batch_size = 64, early_stopping = 5)

        **TA, P, R, F1, train_time, test_time** = evaluate_model(clf, X_test, y_test)

                if **TA** > **best_TA** or (**TA** = = **best_TA** and **train_time** + **test_time** < **best_time**):

            **best_model** = model

            **best_domain** = domain

            **best_TA** = TA

            **best_time** = train_time + test_time

        **endif**

    **endfor**

**endfor**

**best_DTL_model** = best_model

**best_image_domain** = best_domain

**selected_features** = apply_GWO (best_DTL_model, best_image_domain, X_train)

**ONDL** = **train_best_model**(best_DTL_model, selected_features, y_train)

**TA_final, P_final, R_final, F1_final** = evaluate_model(ONDL, X_test, y_test)

**end**

### 4.1 Neutrosophic RGB domain conversion

The concept of neutrosophic logic (NL), was introduced and implemented by Florentin Smarandache [30, 31]. It involves defining three neutrosophic subsets for each event: true (T) value, indeterminacy (I) value, and falsity (F) value. These neutrosophic values (T, I, F) are commonly employed to transform a grayscale image into a neutrosophic image. In this study, we explore the use of neutrosophic RGB for our research, where T represents the waste object, I represent the waste object boundary, and F represents the image background. The image-to-neutrosophic image (NI) conversion is illustrated by Eqs (1)–(4) [32–34]:

$$NI(a,b)=\{T_{a,b},I_{a,b},F_{a,b}\}$$ (1)

$$T_{a,b}=\frac{v(a,b)-v_{min}}{v_{max}-v_{min}}$$ (2)

$$F_{a,b}=1-T_{a,b}$$ 3

$$I_{a,b}=1-\frac{U(a,b)-U_{min}}{U_{max}-U_{min}}$$ (4)

Let *v*(*a*,*b*) is the local mean value of related pixels. *v*_*max*_ and *v*_*min*_ are the maximum and minimum absolute difference pixels of the histogram. *U*(*a*,*b*) is the homogeneity value of *T*(*a*,*b*). While *U*_*max*_ and *U*_*min*_ are the maximum and minimum peaks respectively, measured from *U*(*a*,*b*).

As mentioned earlier, neutrosophic logic is commonly linked to the image grayscale domain. In this study, the researchers utilized the neutrosophic RGB conversion method. The fundamental principle underlying this approach entails the division of the RGB domain into three distinct domains, namely Red, Green, and Blue. Following that, the equations about neutrosophic conversion are implemented autonomously within each respective domain [34]. Ultimately, the images are merged back together within the RGB domain. To obtain a visual depiction of this procedure.

**Fig 3:**
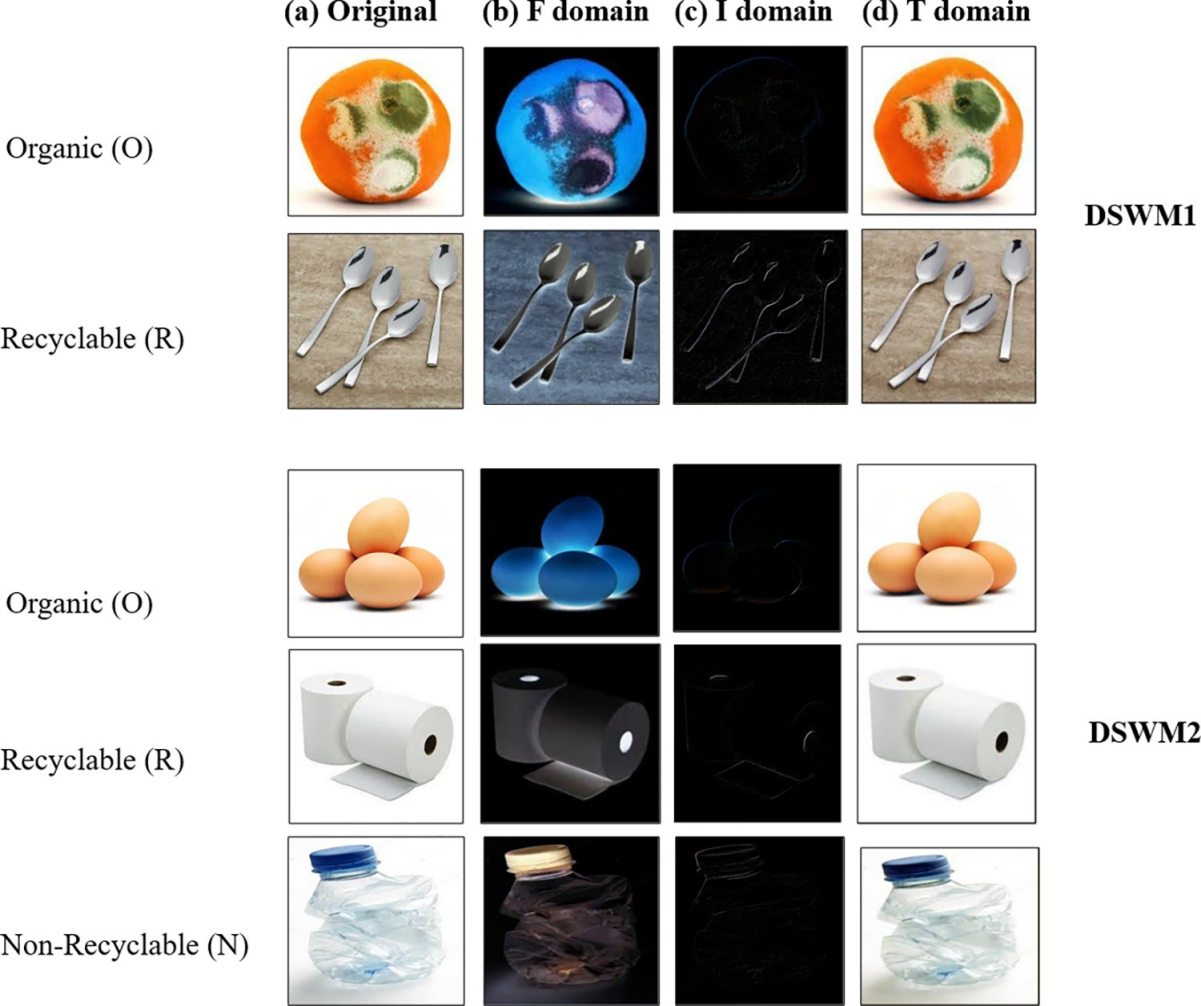
Various neutrosophic RGB image domains were explored in this study, including (a) original RGB images, (b) F domain, (c) I, and (d) T domain for DSWM1 and DSWM2 for the different dataset’s classes.

Fig 3 demonstrates examples of (*T*_*a*,*b*_,*I*_*a*,*b*_,*F*_*a*,*b*_) The images obtained after conducting a neutrosophic image transformation in various domains (T, I, F). Where *T*_*a*,*b*_ the domain of the waste object, *I*_*a*,*b*_ the domain of the waste object’s edges and *F*_*a*,*b*_ the domain of the image background.

### 4.2 Deep Transfer Learning models

The objective of Deep Transfer Learning (DTL) is to mitigate the reliance and expenses associated with training on target data/tasks by leveraging the learned knowledge from a source data/task. Most of the strategies used in Deep Transfer Learning (DTL) are based on network or model architectures. These strategies aim to mitigate the dependence of deep learning models on large quantities of training data and significantly decrease the costs associated with training [35].

**Fig 4:**
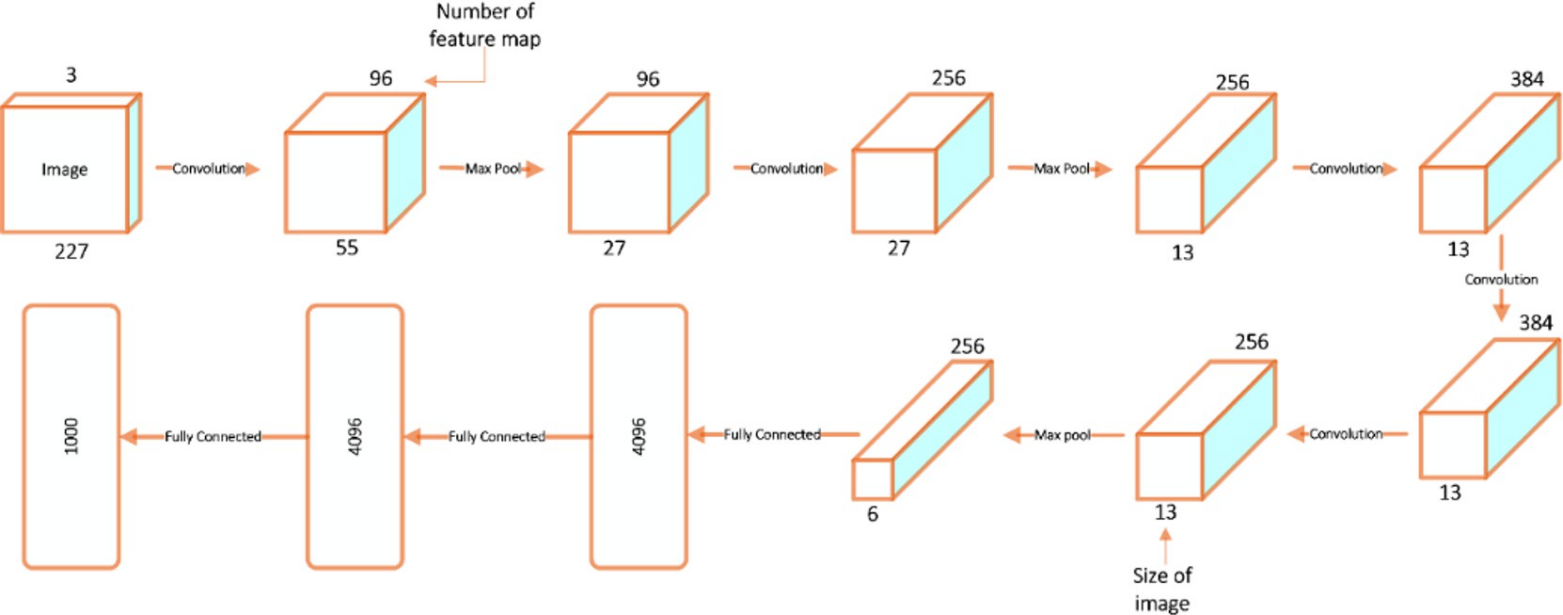
Alexnet architecture.

One of the famous DTL models is Alexnet. It was presented in 2012. It is based on convolution and pooling [36]. AlexNet is one of the first models to use ReLU activation functions, which helps resolve the vanishing gradient problem and accelerated training convergence. Its eight layers, which included five convolutional and three fully connected layers, made it one of the first models to employ ReLU activation functions. Fig 4 depicts the architectural layout.

Another well-known DTL is Googlenet [37], Google developed this revolutionary deep convolutional neural network in 2014. It introduced the concept of "inception" modules, which employ parallel convolutional layers with varying kernel sizes to capture features of varying scales and complexities. The Googlenet architecture consists of 22 layers (including aggregating layers, a total of 27 layers).

Resnet18 [38], was introduced in 2016. It uses shortcut connections to expedite the training of extremely deep networks. By permitting direct information flow across layers, Res-net18 mitigates the problem of vanishing gradients and enables the effective optimization of deeper models. The architecture has 18 layers, including convolutional, batch normalization, max-pooling, and fully connected layers, and is widely used in computer vision tasks.

### 4.3 Grey Wolf Optimization for features selection

The Grey Wolf Optimisation (GWO) [39] was inspired by the leadership and foraging mechanisms of the grey wolf pack, which it simulated. In the wild, the grey wolf is a pack-living canid that resides at the summit of the food chain. The pack adheres to a strict social dominance hierarchy in which the alpha wolf is responsible for the pack’s decision-making, the beta wolf assists the alpha wolf in decision-making, and the delta wolf follows the alpha and beta wolves and dominates the omega wolves. GWO builds such social hierarchy at the beginning of each iteration of the algorithm by assigning three positions with the best fitness values as *α*,*β*, and *δ*, corresponding to the grey wolf pack’s alpha, beta, and delta wolves. Meanwhile, all other positions will then be *ω*, corresponding to the omega wolves.

When a grey wolf pack searches for prey, all wolves will gradually approach the prey and surround it. GWO assumes *α*,*β*, and *δ* have the strongest ability to identify the optimal position and let all *ω* follow them to approach the optimal solution. The procedure is divided into two steps, (1) calculating the distance between each *ω* and *α*,*β*, and *δ*; (2) defining the direction and stride lengths of *ω* to *α*,*β*, and *δ*; (3) updating each *ω*’s position. Eq (5) shows the calculation of the distances between *ω* and *α*,*β* and *δ*.

$$\begin{array}{c} D_{\alpha }=|C_{\alpha }\cdot X_{\alpha }-X_{\omega }|,\\ D_{\beta }=|C_{\beta }\cdot X_{\beta }-X_{\omega }|,\\ D_{\delta }=|C_{\delta }\cdot X_{\delta }-X_{\omega }|, \end{array}$$ (5)

where *D*_*α*_,*D*_*β*_ and *D*_*δ*_ represent the distance between *ω* and *α*,*β* and *δ*, respectively. *X*_*α*_,*X*_*β*_,*X*_*δ*_ and *Xω* represent the positions of *α*,*β*,*δ*, and *ω*, respectively. *C*_*α*_,*C*_*β*_ and *C*_*δ*_ are vectors calculated by Eq (6)

$$C=2\cdot r_{1},$$ (6)

where *r*_1_ is a random vector within the range of [0,1] After calculating the distance between each *ω* and *α*,*β*, and *δ*, the direction and stride lengths of *ω* to *α*,*β*, and *δ* can be calculated by the Eq (7)

$$\begin{array}{c} L_{\alpha }=X_{\alpha }-A_{\alpha }\cdot D_{\alpha },\\ L_{\beta }=X_{\beta }-A_{\beta }\cdot D_{\beta },\\ L_{\delta }=X_{\delta }-A_{\delta }\cdot D_{\delta }, \end{array}$$ (7)

where *L*_*α*_,*L*_*β*_ and *L*_*δ*_ represent the direction and stride lengths of *ω* to *α*,*β*, and *δ*, respectively. *A*_*α*_,*A*_*β*_ and *A*_*δ*_ are vectors calculated by Eq (8).

$$A=2a\cdot r_{2}-a,$$ (8)

where *a* is the convergence factor, decreases linearly from 2 to 0 with iterations going. *r*_2_ is a random vector within the range of [0,1]. The updated position of *ω* can be finally defined by Eq (9).


$$X_{\omega ,new}=\frac{L_{\alpha }+L_{\beta }+L_{\delta }}{3}.$$ (9)

We select GWO as the main feature selection algorithm for its simple design which causes faster convergence rates and less computational overhead, especially in deep learning scenarios that require fast feature selection processes. GWO perform best where high-dimensional data needs to be processed as quickly and efficiently as possible, without adding in any more complexity to the already existing one [40], in order to have a model that may run smoothly but still produce results with high accuracy.

Basically, GWO is convenient at balancing exploration and exploitation a critical ingredient in feature selection tasks [40]. Its mechanism of social hierarchy inspired by alpha, beta, and delta wolves enhances this capability of the algorithm for wide searches in the space of features, refining solutions to get the most relevant features. This balance often leads to better generalization of models that outperforms newer algorithms which may favor one over another. GWO has proven success in image processing tasks[41]; for instance, it is very robust when dealing with high-dimensional feature spaces [42]. Besides other algorithms that may not show this much robustness, this is the rationale for choosing it for this application. GWO reduces dimensionality without sacrificing accuracy; hence, it ensures a highly performing model.

When applied to feature selection, GWO aims to find the most relevant and informative features from a given dataset, thus reducing the dimensionality, and enhancing the performance of machine and deep learning models.

### 4.4 The Optimized Neutrosophic Deep Learning model (ONDL)

**Fig 5:**
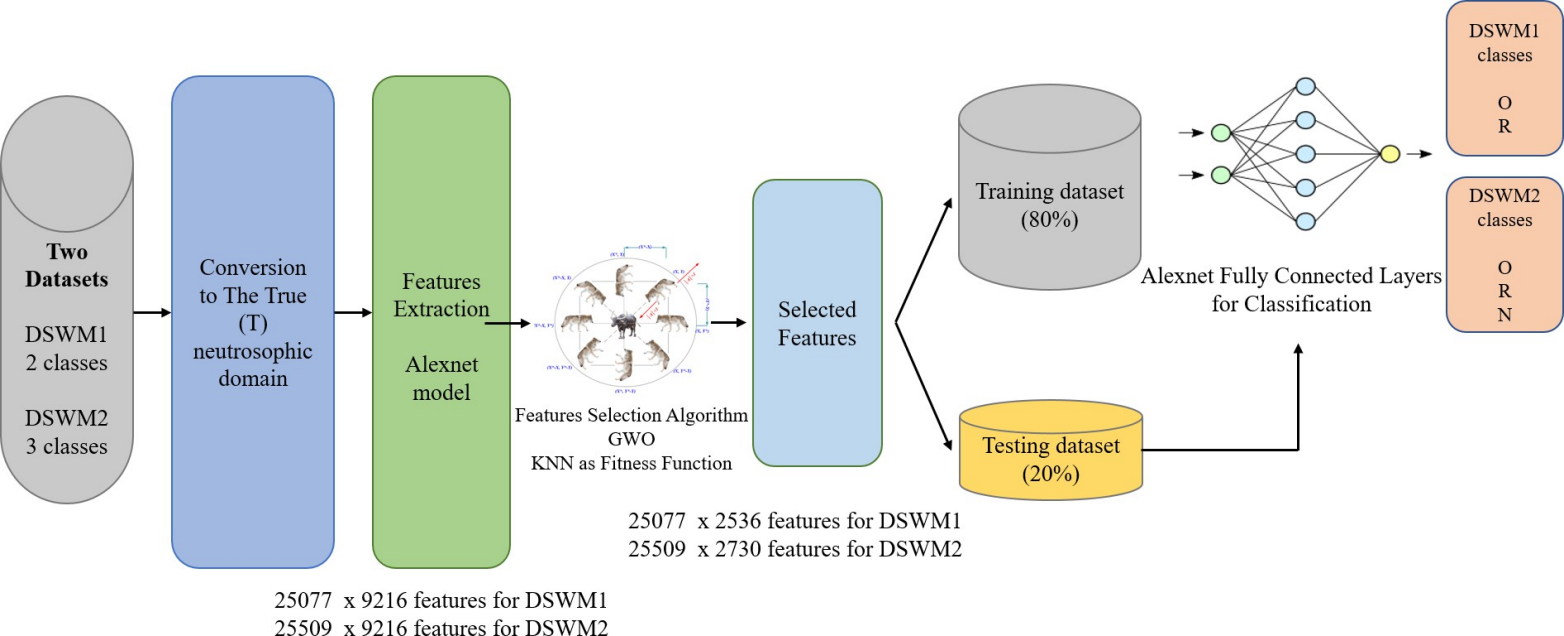
The ONDL design.

The ONDL model’s basic components are introduced in the previous sections. The selection process of the ONDL model among other models is based on the achieved results on testing accuracy and performance metrics that will be introduced in the following section. The ONDL model nominated components are Alexnet for feature generation and classification, The True (T) neutrosophic domain, and GWO for the feature selection. Fig 5 illustrates the ONDEL model design.

The Alexnet component in the ONDL model extracts image features (such as edges, textures, shapes, and complex patterns) using the feature extractor part. The final flattened layer of the Alexnet produces a feature matrix of size N*9216, where N is the number of images in the dataset. This high-dimensional feature matrix serves as the input to the GWO algorithm, which selects a subset of M, the most informative and discriminative feature. The output of GWO is a reduced feature matrix of size N*M, where M is 2536 for DSWM1 and 2730 for DSWM2. The GWO-based feature selection enhances the model’s efficiency and performance by reducing computational complexity, mitigating overfitting, and improving generalization. The reduced feature set also leads to faster testing times, which is crucial in real-world applications, while maintaining high accuracy compared to the original Alexnet model.

## 5 Experimental results and discussion

The first research decision is the selection of the DTL model and the selection of the image domain (original domain or neutrosophic domains). The selection process will be based on the highest achieved accuracy and least consuming time in the training and testing phases. Afterward, GWO will be applied to the selected DTL and image domain to study its effectiveness in detail.

The experiments were piloted on a computer server that was packed with a 2-megahertz Intel Xeon processor as well as 96 GB of random access memory (RAM). As the platform for conducting the numerous experimental trials, MATLAB software was chosen. Throughout the experiments, specific configurations and settings were chosen to ensure consistent and reliable results. To select the DTL and its relevant image domain that will achieve the highest possible testing accuracy and the least amount of time consumed during the training and testing phases, the following configurations and settings were chosen:

- Two Datasets–DSWM1 and DSWM2.
- Four images’ domains:
  ○ The original RGB domain.
  ○ The True (T) neutrosophic domain.
  ○ The Indeterminacy (I) neutrosophic domain.
  ○ The Falsity (F) neutrosophic domain.
- Three DTL models (Alexnet–Googlenet–Resnet18)
- The datasets are divided into two parts [43] (80% of the data for the training process, and 20% for the testing process). The 80/20 split for training and testing is a widely adopted practice in machine learning, providing a sufficient amount of data for training while reserving a reasonable portion for unbiased evaluation
- Data Augmentation [44, 45] was applied to the training data. It helps to increase the diversity of the training data and prevent overfitting, especially given the relatively small size of the datasets.
- The learning rate of DTL models is 0.001. It allows for gradual and stable convergence during training without causing excessive oscillations or getting stuck in local minima.
- The number of epochs to be 50, early stopping [46] to be 5 epochs if the accuracy didn’t improve.
- the mini-batch size [47] is set to 64. It strikes a balance between computational efficiency and gradient stability. It allows for faster training while providing sufficient information for each gradient update.
- Training and testing time and the number of features will be recorded during the experiments.
- The GWO algorithm’s parameters, such as the number of wolves and the maximum number of iterations, were determined through experimentation and fine-tuning to achieve optimal feature selection performance.

- The evaluation criteria selected for performance assessment encompass Testing Accuracy (TA), Precision (P), Recall (R), and F1 Score [33]. The metrics presented in this study are computed utilizing Eqs (10) to (13) and are accompanied by the recorded duration of the training and testing process.

$$Testing\;Accuracy\;(TA)=\frac{TPositive+TNegitive}{\left(TPositive+FPositive)+(TNegitive+FNegitive\right)}$$ (10)

$$Precision\;(P)=\frac{TPositive}{\left(TPositive+FPositive\right)}$$ (11)

$$Recall\;(R)=\frac{TruePositive}{\left(TPositive+FNegitive\right)}$$ (12)

$$F1\;Score=2*\frac{Precision*Recall}{\left(Precision+Recall\right)}$$ (13)

Where TPositive is the total number of True Positive samples, TNegitive is the total number of True Negative samples, FalsePositive is the total number of False Positive samples, and FalseNegitive is the total number of False Negative samples from a confusion matrix.

The empirical findings will be showcased in four discrete subsections. The initial subsection will present a comprehensive analysis of the findings acquired from the first dataset, DSWM1. Subsequently, the subsequent subsection will concentrate on the outcomes derived from the second dataset, DSWM2. In the subsequent section, the results obtained from the implementation of GWO on DSWM1 and DSWM2 will be presented. Finally, the fourth section will provide a comparative analysis between the ONDL model and relevant research studies.

### 5.1 Experimental results for the first dataset (DSWM1)

In this section, the experimental results for DSWM1 will be presented. Table 3 presents the testing accuracy and performance metrics for different DTL models. From the table data, a set of information can be concluded, and they are as follows:

- Alexnet in any image domain achieved the highest testing accuracy possible with its performance metrics except for the (F) domain.
- Alexnet in any image domain achieved the least training and testing time.
- In the falsity (F) domain, the model’s performance is very close to each other. The testing accuracy for the different models is 0.8312, 0.8227, and 0.8371 for Alexnet, Googlenet, and Resnet18 consequently.
- In the falsity (F) domain, the testing accuracy and performance metrics have been improved for Googlenet and Resnet18 models than the original RGB domain.
- Alexnet in the original RGB domain achieved the highest accuracy possible with performance metrics with 0.8578, 0.8593, 0.8558, and 0.8575 in TA, P, R, and F1 consequently.
- In the training and testing time, Alexnet achieved the least consumed time whatever the image domain is.

**Table 3:** Testing accuracy and performance metrics for different DTL models for the first dataset DSWM1.

Model/Metric	TA	P	R	F1	Training time (s)	Testing time (s)
Original RGB Domain						
Alexnet	**0.8578**	**0.8593**	**0.8558**	**0.8575**	968.199083	6.285276
Googlenet	0.8060	0.7960	0.8105	0.8032	1328.791247	7.796522
Resnet18	0.8177	0.8127	0.8168	0.8147	2717.495061	6.38035
True (T) neutrosophic domain						
Alexnet	0.8446	0.8469	0.8424	0.8446	781.159759	**5.938651**
Googlenet	0.7919	0.7805	0.7953	0.7878	1311.579343	7.442283
Resnet18	0.8063	0.7968	0.8081	0.8024	2563.128037	6.667492
Indeterminacy (I) neutrosophic domain						
Alexnet	0.8393	0.8375	0.8366	0.8371	843.637462	5.939909
Googlenet	0.7895	0.7838	0.787	0.7854	1779.088962	7.342477
Resnet18	0.7998	0.7962	0.7968	0.7965	1159.845373	6.168556
Falsity (F) neutrosophic domain						
Alexnet	0.8312	0.8281	0.8288	0.8284	**776.608952**	5.965344
Googlenet	0.8227	0.8164	0.822	0.8192	1325.215742	7.220966
Resnet18	0.8371	0.8346	0.8345	0.8346	989.326114	6.100387

According to the previous information, Alexnet in the original RGB domain and true (T) domain will be selected to apply GWO for the feature selection process for investigation as Alexnet achieved the highest testing accuracy possible with 0.8578 in the original RGB domain and with 0.8446 in the True (T) domain.

### 5.2 Experimental Results for the second dataset (DSWM2)

Table 4 presents the testing accuracy and performance metrics for different DTL models for DSWM2. From the table data, a set of information can be concluded, and they are as follows:

- Alexnet in any image domain achieved the highest testing accuracy possible.
- Alexnet in any image domain achieved the least training and testing time.
- In the True (T), and falsity (F) domains, the testing accuracy of all models has been improved to the original RGB domain.
- Alexnet in the True (T) domain achieved the highest accuracy possible with performance metrics with 0.7751, 0.7068, 0.7097, and 0.7082 in TA, P, R, and F1 consequently.
- In the training and testing time, Alexnet achieved the least consumed time whatever the image domain is.

**Table 4:** Testing accuracy and performance metrics for different DTL models for the second dataset DSWM2.

Model/Metric	TA	P	R	F1	Training time (s)	Testing time (s)
Original RGB Domain						
Alexnet	0.7322	0.6496	0.6761	0.6626	**814.135373**	6.207606
Googlenet	0.7320	0.6757	0.6933	0.6844	2668.084067	8.074914
Resnet18	0.7214	0.6723	0.6747	0.6735	1028.873745	6.259593
True (T) neutrosophic domain						
Alexnet	**0.7751**	**0.7068**	**0.7097**	**0.7082**	950.236882	6.34519
Googlenet	0.7454	0.6802	0.7223	0.7006	1335.560533	7.81629
Resnet18	0.7329	0.6665	0.6888	0.6775	1008.955662	6.355134
Indeterminacy (I) neutrosophic domain						
Alexnet	0.6735	0.5874	0.5809	0.5841	1157.365639	6.19458
Googlenet	0.6728	0.5739	0.6234	0.5977	2402.328079	7.838642
Resnet18	0.6725	0.5955	0.5930	0.5942	2627.879801	6.309215
Falsity (F) neutrosophic domain						
Alexnet	0.7643	0.6970	0.6965	0.6967	1160.805256	**6.149536**
Googlenet	0.7386	0.6793	0.6810	0.6801	1491.740982	7.721447
Resnet18	0.7621	0.6915	0.7184	0.7047	1227.861257	6.353459

According to the previous information, Alexnet in the true (T) domain and the Falsity (F) domain will be selected to apply GWO for the feature selection process for investigation as Alexnet achieved the highest testing accuracy possible with 0.7751 in the True (T) domain and with 0.7643 in the Falsity (F) domain.

### 5.3 Experimental results of GWO for the DSWM1 and the DSWM2

In sections 5.1 and 5.2, The result of the selection process ended up with selecting Alexnet as the main model for the investigation as it achieved the highest testing possible whatever the domain was, or the dataset was.

**Fig 6:**
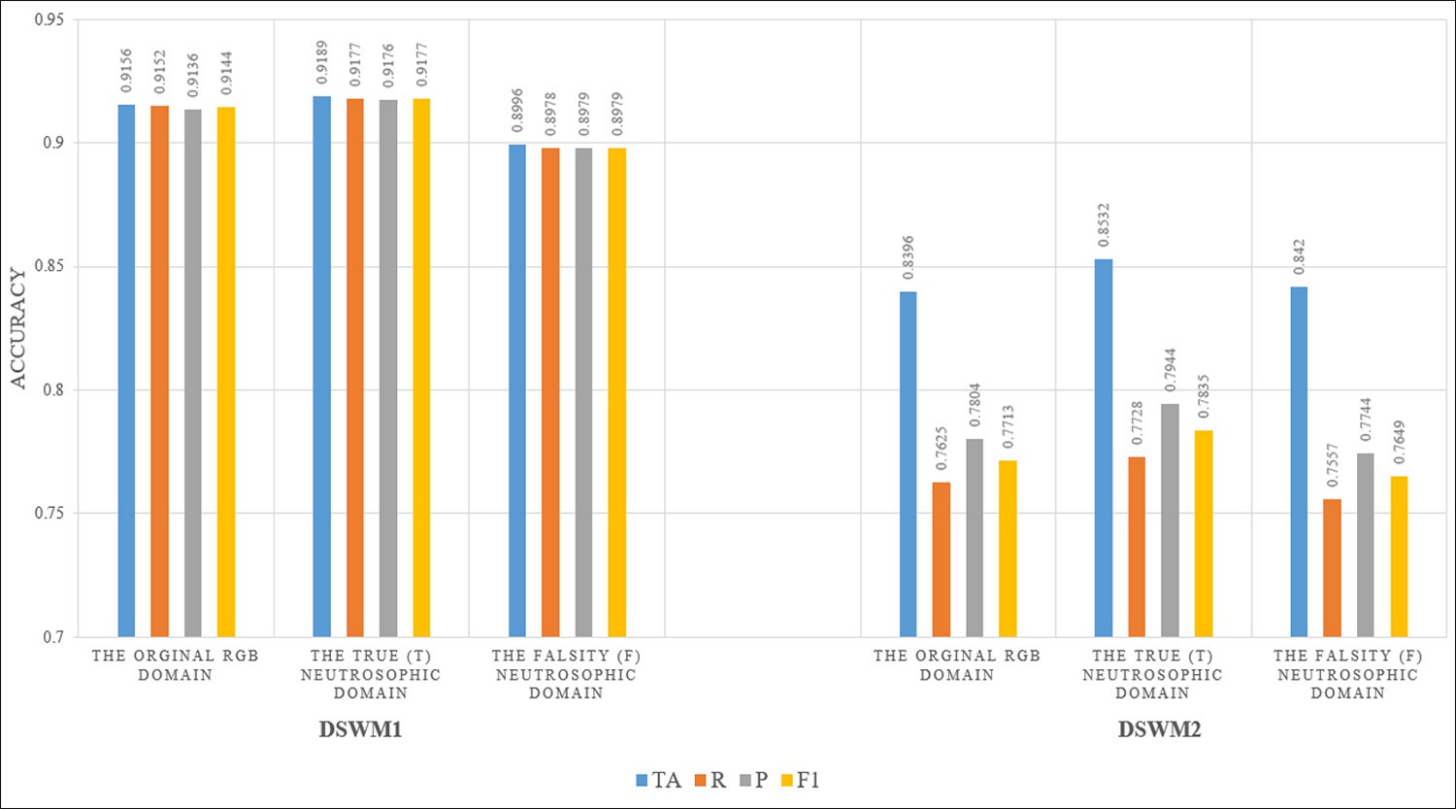
Testing accuracy and performance metrics for Alexnet in the original RGB, True (T) neutrosophic, and Falsity (F) neutrosophic domains after feature selection using GWO for DSWM1 and DSWM2.

In this section, the Alexnet model will be investigated after the feature selection process using GWO. The image domains that will be investigated are the Original RGB Domain, the True (T) neutrosophic domain, and the Falsity (F) neutrosophic domain for the DSWM1 and the DSWM2. Fig 6 illustrates the Testing accuracy and performance metrics for Alexnet in the original RGB, True (T) neutrosophic, and Falsity (F) neutrosophic domains after feature selection using GWO.

From Fig 6, some interesting facts can be deduced which will be the core design of the proposed model in this research (the ONDL model). The facts are as follows:

- The highest accuracy possible was achieved in the True (T) neutrosophic domain whether the dataset was DSWM1 or DSWM2. The ONDL model achieved 0.9189 testing accuracy in DSWM1 and 0.8532 in DSWM2.
- The achieved testing accuracy of the ONDL model is supported by performance metrics. In DSWM1, the performance metrics achieved 0.9177, 0.9176, and 0.9177 in P, R, and score. In DSWM2, the performance metrics achieved 0.7728, 0.7944, and 0.7835 in P, R, and F1 score.

**Fig 7:**
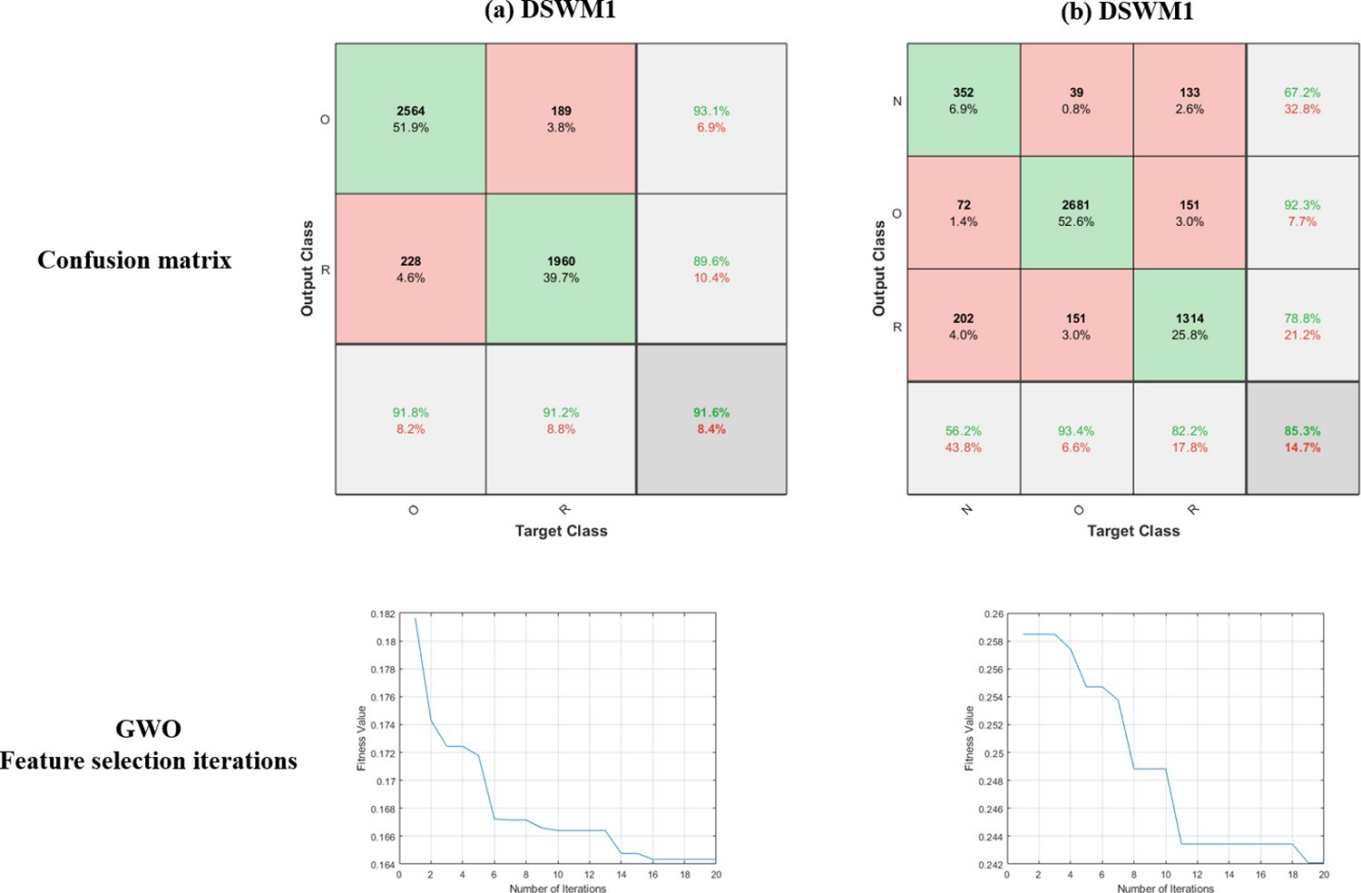
Confusion matrix and Feature selection iteration for GWO for the ONDL model for the dataset (a) DSWM1, and (b) DSWM2.

The ONDL model performance metrics were calculated based on the confusion matrix. The confusion matrix for the ONDL model is presented in Fig 7. Fig 7 presents the accuracy for every class for the ONDL model. In DSWM1, the class accuracy for the (O) class is 0.931, while for the (R) class is 0.896. In DSWM2, the class accuracy for the (O) class is 0.923, while for the (R) class is 0.788 and for the (N) class is 0.672. Also, Fig 7 presents the progress of iterations for the GWO algorithm for selecting the best features for the ONDL model for DSWM1 and DSWM2.

The ONDL model was selected and nominated during the selection process. It proved its effectiveness in the classification task whatever the dataset is. The ONDL model achieved the highest accuracy possible if it is compared to the original model Alexnet in the original domain without optimization. Table 5 presents the ONDL model against the original Alexnet in the RGB domain without optimization for the DSWM1 or DSWM2.

**Table 5:** The ONDL model against the original Alexnet in the RGB domain without optimization for the DSWM1 or DSWM2 with training, testing time, and number of features.

Model/Metric	TA	P	R	F1	FS and Training time (s)	Testing time (s)	# Features
DSWM1							
Alexnet	0.8578	0.8593	0.8558	0.8575	968.19901	6.485276	9216
ONDL	0.9189	0.9177	0.9176	0.9177	1965.6715	2.48905	2536
DSWM2							
Alexnet	0.7322	0.6496	0.6761	0.6626	814.13537	6.207606	9216
ONDL	0.8532	0.7728	0.7944	0.7835	2116.7255	2.69515	2730

Table 5 illustrates that the ONDL model improves the classification testing accuracy by 6.11% over the Alexnet on the DSWM1. On DSWM2, the ONDL model also improves the classification testing accuracy by 12.1%. Moreover, the performance metrics in both datasets have also been improved. One of the drawbacks of the ONDL model is the time consumed in the feature selection process. The consumed time for the feature selection and training is doubled than the Alexnet. However, in deep learning research, the training time can be neglected. The testing time is crucial in deep learning, the ONDL model achieved less time than Alexnet in the testing phase. The less testing time is due to the number of selected features as ONDL reduces the number of features for the testing. The ONDL model selected 2536 features in DSWM1 and 2730 features in DSWM2 while Alexnet works on 9216 features.

### 5.4 The ONDL model VS related works

One of the methods to evaluate the ONDL model is the comparison with related works that used the same datasets. Table 6 presents a comparison of the ONDL model and the other related works. The ONDL model achieved competitive results with related works in terms of testing accuracy. It achieved 91.89% in testing accuracy in DSWM1 while related works achieved 80.88% in [26] and 88.7% in [17]. In DSWM2, the ONDL model achieved 85.32% in testing accuracy while in [27], their proposed CNN model achieved 81.25%.

**Table 6:** A comparison of the ONDL model and the other related works.

Model/Metric	Description	TA
DSWM1		
[26]	Proposed a five-layer CNN	80.88%
**ONDL (Ours)**	Alexnet + True (T) neutrosophic domain + GWO	91.89%
DSWM2		
[27]	Proposed a CNN Model	81.25%
**ONDL (Ours)**	Alexnet + True (T) neutrosophic domain + GWO	85.32%

The superior performance of the ONDL model can be attributed to several points, first, the conversion of RGB images to the True (T) neutrosophic domain enhances the representation of waste objects by focusing on the object itself while suppressing background noise and edge ambiguity. Second, the incorporation of GWO for feature selection plays a crucial role in enhancing the model’s performance. By identifying and retaining the most salient features, GWO reduces the dimensionality of the feature space, mitigating overfitting and improving generalization. The combination of Alexnet for feature extraction and GWO for feature selection creates a synergistic effect. The deep learning model leverages its pre-trained knowledge to capture complex patterns in the images, while GWO refines the feature set by selecting the most informative ones. This synergy allows the ONDL model to achieve high accuracy and efficiency in waste classification.

The study presented in [17] utilizes the DSWM1 dataset and reports a testing accuracy of 94.53% and a precision of 88.70%. The proximity of the Testing Accuracy (TA), Precision (P), Recall (R), and F1-score values in the proposed ONDL model (91.89%, 91.77%, 91.76%, and 91.77%, respectively) indicates its robustness. While the model in [17] surpasses the ONDL model in terms of testing accuracy by 2.64%, the ONDL model exhibits a superior precision with a 3.07% improvement. The emphasis on precision is particularly crucial in waste management, as false positives (non-recyclable items incorrectly classified as recyclable) can lead to contamination of recycling streams, process disruptions, and increased costs. The ONDL model’s high precision ensures stringent classification, minimizing such errors and promoting efficient recycling efforts. By prioritizing precision, the ONDL model contributes to resource conservation and a more sustainable waste management system by accurately identifying and diverting recyclable materials from landfills.

Deeper DTL models such as Nasnet-large [48], Densenet-201 [49], and Inception-resnet-v2 [50] can achieve better testing accuracy than the ONDL model. Those models had large numbers of layers, over 150 layers in their architecture. They had the advantage of extracting better features from images which would be reflected in the achieved testing accuracy. The ONDL model had 25 layers and was constructed upon the Alexnet which is considered one of the smallest architectures in DTL models. It is an advantage; ONDL will consume less training time, testing time, memory, and computation. The ONDL model can fit to run on mobile and IoT devices while deeper DTL models can’t.

## 6 Conclusion and future works

Waste management plays a critical role in the achieving of sustainability goals, as it endeavors to confront the complicated challenges arising from the generation and disposal of waste, including ecological, social, and economic challenges. Sustainable waste management practices seek to minimize waste generation, promote recycling and reuse, and ensure proper disposal of residual waste to reduce environmental pollution and conserve natural resources. In this paper, An Optimized Neutrosophic Deep Learning (ONDL) model was proposed to classify waste objects. Two datasets were tested in this research. The first Dataset for Waste Management (DSWM1) included two classes (Organic or Recycled) objects. The Second Dataset for Waste Management (DSWM2) included three classes (Organic, Recycled, or Non-Recyclable) objects. The ONDL model architecture was based on Alexnet as a deep transfer learning model and the conversion of images to True (T) neutrosophic domain and GWO for feature selection. The selection process of the building components of the ONDL model was comprehensive as different DTL models (Alexnet, Googlenet, and Resnet18) were tested, and three neutrosophic domains (T, I, and F) domain were included. The ONDL model proved its effectiveness against all the tested models, moreover, it achieves competitive results with related works in terms of testing accuracy and performance metrics such as P, R, and F1 score. In DSWM1, the ONDL model achieved 0.9189, 0.9177, 0.9176, and 0.9177 in TA, P, R, and F1 Score. In DSWM2, it achieved 0.8532, 0.7728, 0.7944, and 0.7835 in TA, P, R, and F1 Score. One of the limitations of the ONDL model is the out-of-distribution data (unseen or novel varieties of new waste objects), it may struggle to classify those varieties accurately. The study’s findings are subject to certain threats to validity. The use of publicly available datasets, while beneficial for reproducibility, may introduce biases or limitations inherent to the data collection process. Also, the study makes certain assumptions. It assumes that the images in the datasets are representative of real-world waste objects and that the labels assigned to them are accurate. The model’s performance might vary when applied to real-world waste streams with different characteristics or distributions. There are several potential areas for future research on the ONDL model for waste classification. The investigation of GWO variants, such as the improved GWO (IGWO), the binary GWO (BGWO), or the hybrid GWO with other optimization techniques, to the ONDL model. One possibility is to investigate the impact of different optimization algorithms such as the Whale Optimization Algorithm (WOA), the Salp Swarm Algorithm (SSA), or the Harris Hawks Optimization (HHO) algorithm which are not investigated in this research for feature selection techniques to further improve model accuracy and efficiency. Additionally, it would be interesting to compare the performance of the ONDL on different datasets rather than the datasets investigated in this research.
